# Network Analysis of Parental-Economic Factors and Symptoms of Suicidal Ideation Among Left-Behind Children in Unprivileged Regions in China

**DOI:** 10.31083/AP43496

**Published:** 2025-06-10

**Authors:** Yang Yu, Qianyu Zhang, Xuerong Liu, Mengjie Luo, Xiaolin Zhang, Xianyong An, Jingxuan Zhang

**Affiliations:** ^1^School of Psychology, Army Medical University, 400038 Chongqing, China; ^2^School of Basic Medicine, Army Medical University, 400038 Chongqing, China; ^3^Teaching Examination Center, Army Medical University, 400038 Chongqing, China; ^4^Department of Rehabilitation Medicine, Nanchong Psychosomatic Hospital, 637700 Nanchong, Sichuan, China

**Keywords:** left-behind children, suicidal ideation, parental-economic factors, network analysis, preventive measures

## Abstract

**Objective:**

This study aimed to investigate relationships between parental-economic factors and suicidal ideation among left-behind children in underprivileged regions of China using network analysis, to pinpoint key factors and pathways contributing to suicidal ideation, thereby facilitating evidence-based suicide preventive interventions.

**Methods:**

In total, 1076 left-behind children were selected from a large dataset (N = 249,772) after applying exclusion criteria. Suicidal ideation was assessed via the Positive and Negative Suicide Ideation Inventory-Chinese Version (PANSI-C). The outcomes were grouped into positive suicidal ideation and negative suicidal ideation within the network analysis framework. Sociodemographic data, parental status, and economic status were also recorded. Through network analyses, centrality and bridge indices were calculated. Network stability and accuracy were evaluated by bootstrapping methods.

**Results:**

The network had three communities: positive suicidal ideation, negative suicidal ideation, and covariates. Strong positive correlations were observed within communities, especially among “life worth”, “confident”, and “satisfy”. Nodes “failure”, “lonely and sad”, “confident”, and “satisfy” exhibited the highest expected influence. Nodes “hopeless”, “life worth”, and “satisfaction of family members’ relationships” served as bridges between the covariates and suicidal ideation. Significant structural differences existed between female and male networks.

**Conclusions:**

This study highlights the multifaceted nature of suicidal ideation among left-behind children, which is influenced by various parental-economic factors. Key node and bridge links offer targets for tailored interventions. Gender-sensitive approaches are imperative in suicide preventive measures. Network analysis provides a comprehensive framework to unravel complex relationships, informing evidence-based interventions for left-behind children.

## Main Points

1. The study revealed a complex network of relationships between suicidal ideation and parental-economic factors among left-behind children, highlighting the multifaceted nature of suicidal ideation and how it is influenced by a series of family economic factors.

2. Certain nodes in the network of suicidal ideation, such as “failure”, “lonely and sad”, “confident”, and “satisfy”, play influential roles in the progression of suicidal ideation. Additionally, the subjective attitude of “satisfaction of family members’ relationships” was identified as a key factor in preventive strategies among all the parental-economic factors.

3. This study underscores the significance of adopting gender-sensitive approaches in suicide preventive measures. In female networks, satisfaction regarding family members’ relationships is more closely linked to suicidal ideation, suggesting that females are more sensitive to family relationships. These findings indicate that the patterns of relationships between parental-economic factors and suicidal ideation symptoms vary between females and males.

## 1. Introduction

In recent years, the issue of left-behind children (LBC) in China has garnered significant attention from researchers and the public. The rapid pace of urbanization and economic growth has led to a mass migration of individuals from rural to urban areas, frequently resulting in children being left behind in rural regions under the care of extended family or other relatives. This phenomenon has been associated with a multitude of psychological and social challenges for these children, including an increased risk of suicidal ideation [1, 2]. Parental migration and economic factors are the principal drivers of LBC issues, and they have been studied extensively in the past [3, 4, 5, 6, 7, 8]. However, the multifaceted nature of these factors—parental migration, economic conditions, and suicidal ideation—introduces complexities in understanding their intricate relationships and relative significance.

Multiple studies have highlighted the adverse effects of parental migration on LBC’s mental health [3, 6, 9]. A systematic review and meta-analysis conducted by Fellmeth *et al*. [3] across 16 countries, including China, found that parental migration negatively impacts LBC’s mental health, particularly in terms of depression, anxiety, and suicidal ideation. Ma *et al*. [4] specifically probed the correlations between Chinese parents’ labor migration and their offspring’s engagement in non-suicidal self-injury (NSSI) and suicidal ideation, concluding that the migration of fathers or both parents significantly correlates with an augmented risk of NSSI in children. Furthermore, whether it is single or both parental migration [4, 5, 10], the initial time of migration [4] and its duration [11] have been identified as pivotal factors in suicide-related behaviors. From another perspective, some researchers have reported that family function [1, 12, 13, 14, 15, 16] plays an important role in LBC’s suicidal thoughts or behaviors beyond migration itself. Additionally, the subjective feelings toward family functions [17, 18], parent-child attachment [5], and member relationships [14] also influence suicide in LBC. According to these findings, the factors of family function and the relationships between parents and children (including subjective feelings and objective status) can partly explain how parental factors influence children’s suicidal ideation. This may illustrate the sociopsychological mechanism of the effects of parental factors, which has been considered rarely in previous studies.

Economic status, another critical factor, has been found to impact the mental health of LBC. It has been established that in China the economic status of a family is equivalent to the economic status of the parents [19, 20]. Numerous studies have documented the adverse effects of poverty and economic hardship on mental health [21, 22]. Low socioeconomic status (SES) has been associated with heightened stress, anxiety, and depression, ultimately exacerbating suicidal ideation [23]. Additionally, the absence of economically stable parents further compounds these risks, as parents may face financial insecurity and limited access to resources and support [24]. Specifically, Mahumud *et al*. [18] assessed the global burden of suicidal behaviors among adolescents in 77 low- and middle-income countries and reported that a lack of economic resources and poverty were significant risk factors for suicidal behaviors. In line with this, Jeong’s study [25] on South Korean adolescents revealed a U-shaped relationship between suicidal ideation and both perceived stress levels and family economic status, indicating that intermediate levels of stress or economic status minimize the likelihood of suicidal ideation, whereas higher or lower levels exacerbate it. Economic status influences adolescents through the mediatory effects of parents’ mental health and the adverse effects on their parenting styles [26, 27]. The family stress model (FSM) suggests that financial struggles and pressures within families indirectly shape children’s adjustment by impacting parents’ behavioral and emotional responses [28]. This finding implies that children’s perceptions of economic status may elucidate how it affects their mental well-being, including suicidal ideation. Although the above studies have shown that economic status significantly affects suicide, research has yielded inconsistent findings regarding the direction and magnitude of these effects. Moreover, studies examining subjective economic status are scarce, necessitating further investigation into the intricate relationships between economic factors and suicide-related behaviors.

In previous studies, Beck’s Scale for Suicide Ideation (BSI) [29, 30] has been widely used among adults. However, the BSI cannot discriminate between attempters and ideators [30]. Except for the items representing suicide behaviors, the remaining items form a single factor structure [30], which includes all negative descriptions. Other studies have discovered the multifactorial structure of suicidal ideation, identifying different kinds of dimensional patterns, including “passive and active ideation” [31], “passive and active ideation with three other facets” [32], “suicide desire, and resolved plans and preparations” [33], “minor suicidal ideation, specific plans and desires for suicide, and morbid ideation” [34], etc. The ideation measured in the aforementioned studies is also described as extending beyond suicidal thoughts to include plans or behavioral intentions, which means these measurements cannot focus solely on thoughts. The Positive and Negative Suicidal Ideation Inventory (PANSI) serves as another instrument for evaluating suicidal ideation, characterized by its dual-factor composition, incorporating both positively and negatively described items, which is more suitable for children and adolescents [35]. The PANSI has been shown to be reliable among children and adolescents across different cultures [35, 36, 37]. In contrast to other measurements, its items focus exclusively on thoughts, thereby enabling a concentrated examination of the influences on suicidal ideation, rather than broader suicide constructs. This targeted focus is crucial for preventing suicide in LBC as early as possible.

Given the multifaceted nature of this problem, network analysis offers a comprehensive framework [38] to examine the intricate relationships between parental-economic factors and suicidal ideation among LBC. This approach enables the identification of key nodes (i.e., observable variables rather than latent structures) and also elucidates their interconnectivity [38], thereby providing insights into the underlying mechanisms and pathways that contribute to suicidal ideation. For example, network analysis can reveal how different economic statuses, parental migration factors, subjective attitudes towards family relationships, and other demographic variables influence suicidal ideation, allowing for the development of targeted preventive interventions.

Therefore, this study aimed to utilize network analysis to investigate the complex interactions between parental-economic factors and suicidal ideation among LBC in China. By shedding light on the intricate web of factors contributing to this pressing issue, this study sought to inform evidence-based preventive intervention strategies aimed at mitigating the mental health burden faced by LBC. By addressing the root causes, the sociopsychological mechanism, and key factors, we can elucidate how parental-economic factors work through subjective family function and economic status to foster healthier and more resilient environments for these vulnerable children.

## 2. Materials and Methods

### 2.1 Sampling

#### 2.1.1 Dataset Acquisition

The participants were selected from a large-scale and multicenter cohort dataset (N = 249,772) [39] on the effects of psychological care on depression and suicidal ideation in underrepresented children. This dataset was compiled by the Psychological Health Guard for Children and Adolescents Project of China (CPHG) Group. The surveys were conducted by Nanchong Psychosomatic Hospital and North Sichuan Medical College.

#### 2.1.2 Data Inclusion and Exclusion Criteria

We set inclusion and exclusion criteria to avoid bias, which ensured that results could be generalized.

The inclusion criteria were as follows: aged 12 to18 years, completed the survey of suicidal ideation, and provided demographic information.

The exclusion criteria were as follows: had a chronic history of psychiatric diseases and had a chronic history of drug use.

We used baseline data (n = 35,065) to conduct cross-sectional analyses. A total of 34,666 participants completed the survey of suicidal ideation and were aged 12 to 18 years, among which 1169 participants provided demographic information. Then we excluded those who had a chronic history of psychiatric diseases or drug use, resulting in 1076 valid cases (379 males, 697 females; average age = 14.43 years, SD = 1.65) for analysis.

### 2.2 Measurements

#### 2.2.1 Suicidal Ideation

Suicidal ideation was measured using the Positive and Negative Suicide Ideation Inventory-Chinese Version (PANSI-C), which was developed by Osman *et al*. [40] specifically for children and adolescents. The Chinese version was translated by Chang *et al*. [41] The PANSI-C includes 14 items (scores ranging from 14 to 70), with six items related to positive suicidal ideation (PSI, scores ranging from 6 to 30) and eight items related to negative suicidal ideation (NSI, scores ranging from 8 to 40). In this study, the PANSI-C was reliable, with a Cronbach’s alpha equal to 0.892. The Cronbach’s alpha values of the PSI and NSI were 0.867 and 0.929, respectively.

#### 2.2.2 Sociodemographic Data

Sociodemographic information, including gender (i.e., female/male), age, only child status (i.e., only child/non-only child), and residence (i.e., city/town/rural areas), was collected from the registration systems of the schools and confirmed by the child and adolescents.

#### 2.2.3 Parental Status

The parental status mainly consisted of several items regarding the experience of separating from parents, including living status with parents (i.e., living with both parents/living with one parent/living with other relatives/living in the social welfare institute), age at separation (i.e., 0~1.5/1.5~3/3~6/6~12/12~18 years), and years of separation (i.e., 0.5~1/1~2/2~4/4~10/above 10 years). Additionally, their attitudes toward the family relationships were measured using a 5-point Likert scale (i.e., very satisfied/relative satisfied/middle level/relatively dissatisfied/very dissatisfied). Higher scores represent more negative attitudes.

#### 2.2.4 Economic Status

Economic status included two items, one of which measured the objective family annual income (i.e., RMB below 60,000/60,000~150,000/150,000~300,000/above 300,000; The exchange rate: 1 USD = 7.19 RMB; 1 EUR =7.59 RMB respectively, as of December 2024) and the other measured the participants’ subjective feelings (i.e., poor/normal/middle/rich) of the family economic status.

### 2.3 Statistical Analyses

#### 2.3.1 Descriptive Statistics

Demographic characteristics and descriptive statistics were analyzed using IBM SPSS version 25.0 (IBM Corp., Armonk, NY, USA) [42].

#### 2.3.2 Network Analyses

Network analyses were conducted using R 4.4.1 (R Foundation for Statistical Computing, Vienna, Austria) [43] in RStudio 2024.04.2 (Boston, MA, USA) [44]. We used several R packages to perform network estimation, centrality and bridge index analyses, stability and accuracy analyses, and network comparisons.

We included all the continuous and ordinal variables in a network. Then a Gaussian graphical model (GGM) [45] was applied to estimate the network structure, utilizing a partial correlation model (PCM) [45] for edge weight estimation and the graphic least absolute shrinkage and selection operator (GLASSO) algorithm [46] to shrink weak correlations to zero. These methods led to a stable network. The network estimation was conducted using the R “qgraph” package [47], which included the “EBICglasso” function for the GLASSO algorithm and a visualization function to display the network structure in a picture.

Centrality, the core index of network analyses, refers to the degree of correlation of one node with others within a network [47]. We built a weighted network in this study and used “strength”, “closeness”, and “betweenness” as the centrality indices. “Strength” represents the sum of the weights of all the edges of one node. “Closeness” and “betweenness” can be used in both weighted and unweighted networks, which are less stable than “strength” in weighted networks [48]. “Closeness” is the sum of the shortest paths of one node with all other nodes, whereas “betweenness” is the degree of one node’s bridge function within a network [49]. Additionally, we calculated a more appropriate index, “expected influence”, instead of “strength” when the edges included both positive and negative edges [50]. The psychometric properties showed that the PANSI-C consists of two factors, specifically, positive and negative suicidal ideation [37]. The covariates representing parental-economic factors and residence in sociodemographic factors are ordinal, whereas gender and only child status are dichotomic variables. Therefore, we included parental-economic factors, residence, and age (which are ordinal and continuous variables) in the covariate community and considered positive and negative ideation as two additional communities. We then considered bridge indices [51] to estimate the most important variable linking the covariates and positive/negative suicidal ideation. The bridge indices also include strength and expected influence, named “bridge strength” and “bridge expected influence” [52]. In R, we applied the “networktools” package [53] to calculate bridge indices.

Because of the possible instability and unreliability of the network, we conducted post-hoc stability and accuracy analyses using the R “bootnet” package [54]. We used bootstrapping methods to calculate the 95% confidence intervals (CI) for the accuracy of the edge weights. The correlation stability (CS) coefficient calculated using case-dropping bootstrapping methods could also represent the edge weight accuracy, for which the recommended value was not less than 0.5 [54]. We also used the CS coefficient to represent the centrality stability, which is recommended to be not less than 0.25 (better than 0.5) [54].

Gender and only child status were not included in the network. We took them as classified variables and conducted network comparisons using the R “NetworkComparisonTest” package [55] to test the network variance and the global strength variance between different groups (i.e., female/male, only child/non-only child).

## 3. Results

### 3.1 Demographic Characteristics and Descriptive Statistics

Demographic information, descriptive statistics of positive/negative suicidal ideation, and covariates are shown in Table 1.

**Table 1.** **Demographic information and descriptive statistics**.

Variable		N (1076)	%	Mean ± SD	Median (IQR)
Gender					
	Female	697	64.78		
	Male	379	35.22		
Age (years)				14.43 ± 1.65	
Only child status					
	Only child	194	18.03		
	Non-only child	882	81.97		
Residence					2.00 (1.00)
	City (1)	527	48.98		
	Town (2)	317	29.46		
	Rural areas (3)	232	21.56		
Living status with parents					2.00 (1.00)
	Living with both parents (1)	254	23.61		
	Living with one parent (2)	424	39.40		
	Living with other relatives (3)	398	36.99		
	Living in the social welfare institute (4)	0	0		
Age at separation (years)					3.00 (2.00)
	0~1.5 (1)	177	16.45		
	1.5~3 (2)	205	19.05		
	3~6 (3)	203	18.87		
	6~12 (4)	324	30.11		
	12~18 (5)	167	15.52		
Duration of separation (years)					3.00 (3.00)
	0.5~1 (1)	336	31.22		
	1~2 (2)	135	12.55		
	2~4 (3)	191	17.75		
	4~10 (4)	241	22.40		
	>10 (5)	173	16.08		
Attitude to family relationships					3.00 (1.00)
	Very satisfied (1)	168	15.61		
	Relative satisfied (2)	367	34.11		
	Middle level (3)	380	35.32		
	Relatively dissatisfied (4)	119	11.06		
	Very dissatisfied (5)	42	3.90		
Family annual income (RMB Yuan)					1.00 (1.00)
	<60,000 (1)	647	60.13		
	60,000~150,000 (2)	356	33.09		
	150,000~300,000 (3)	61	5.67		
	>300,000 (4)	12	1.11		
Feelings of family economic status					2.00 (0.00)
	Poor (1)	231	21.47		
	Normal (2)	683	63.48		
	Middle (3)	160	14.87		
	Rich (4)	2	0.18		
PANSI-C				33.54 ± 8.67	
PSI				19.08 ± 4.73	
NSI				14.47 ± 5.97	

Note: N, number of valid samples; IQR, interquartile range; PANSI-C, Positive and Negative Suicide Ideation Inventory-Chinese Version; PSI, positive suicidal ideation; NSI, negative suicidal ideation; SD, standard deviation. “(1), (2), (3), (4), (5)” represent the code of different options of the variables. The exchange rate: 1 USD = 7.19 RMB; 1 EUR =7.59 RMB respectively, as of December 2024.

### 3.2 Network Structure

**Fig. 1.**
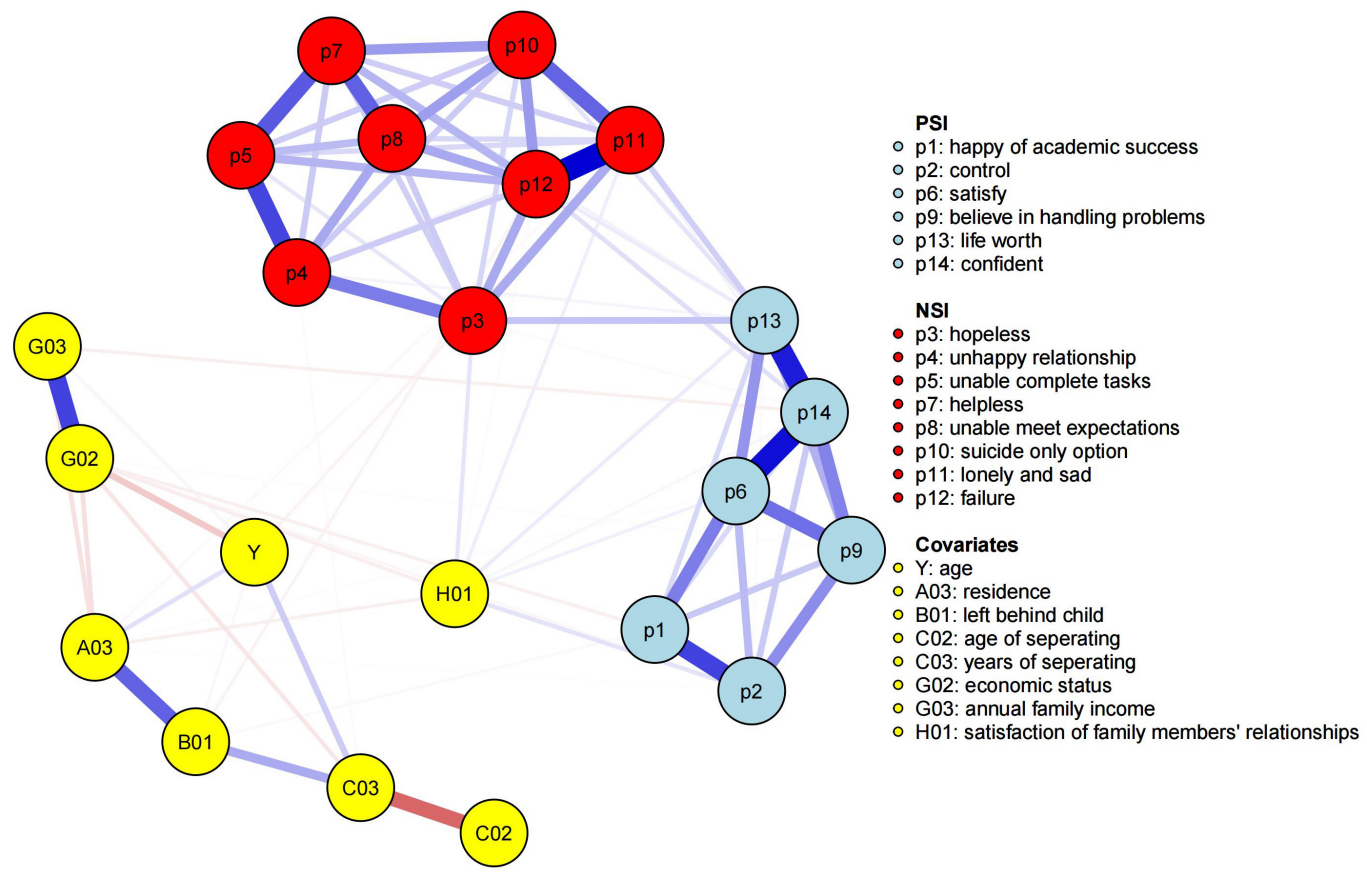
**Graphical structure of the network**. Different colors of nodes belong to different communities. The blue edges represent positive weighted correlations, whereas the red edges represent negative weighted correlations. The darker the edge color is, the higher the edge weight is.

The network was structured as three communities: positive suicidal ideation, negative suicidal ideation, and covariates (see Fig. 1). There were 84 nonzero edges out of a total of 231 possible edges. The edges “p13—p14” (“life worth—confident”), “p6—p14” (“satisfy—confident”), and “p11—p12” (“lonely and sad—failure”) had weights of 0.33, 0.35, and 0.37, respectively. These five nodes (“p6”, “p11”, “p12”, “p13”, and “p14”) had relatively strong positive linkages (weighted above 0.30) within their communities, which indicated that these nodes and edges were core symptom groups of positive or negative suicidal ideation. Moreover, within the whole community of suicidal ideation (including PSI and NSI), nodes “p3” (“hopeless”), “p11” (“lonely and sad”), “p12” (“failure”), and “p13” (“life worth”) all had more than nine nonzero edges (more than two-thirds of the 13 edges) linked with other nodes. The number of edges linked to “p13” was 11, which was the greatest. The results indicated that the aforementioned nodes might exert greater influence within the suicidal ideation structure. Among all the nodes in the covariate community, “H01” (“satisfaction of family members’ relationships”) was closely associated with the PSI and NSI communities, which had seven linkages with the nodes from suicidal ideation. The values of the edge weights are shown in **Supplementary Table 1**.

### 3.3 Centrality and Bridge

**Fig. 2.**
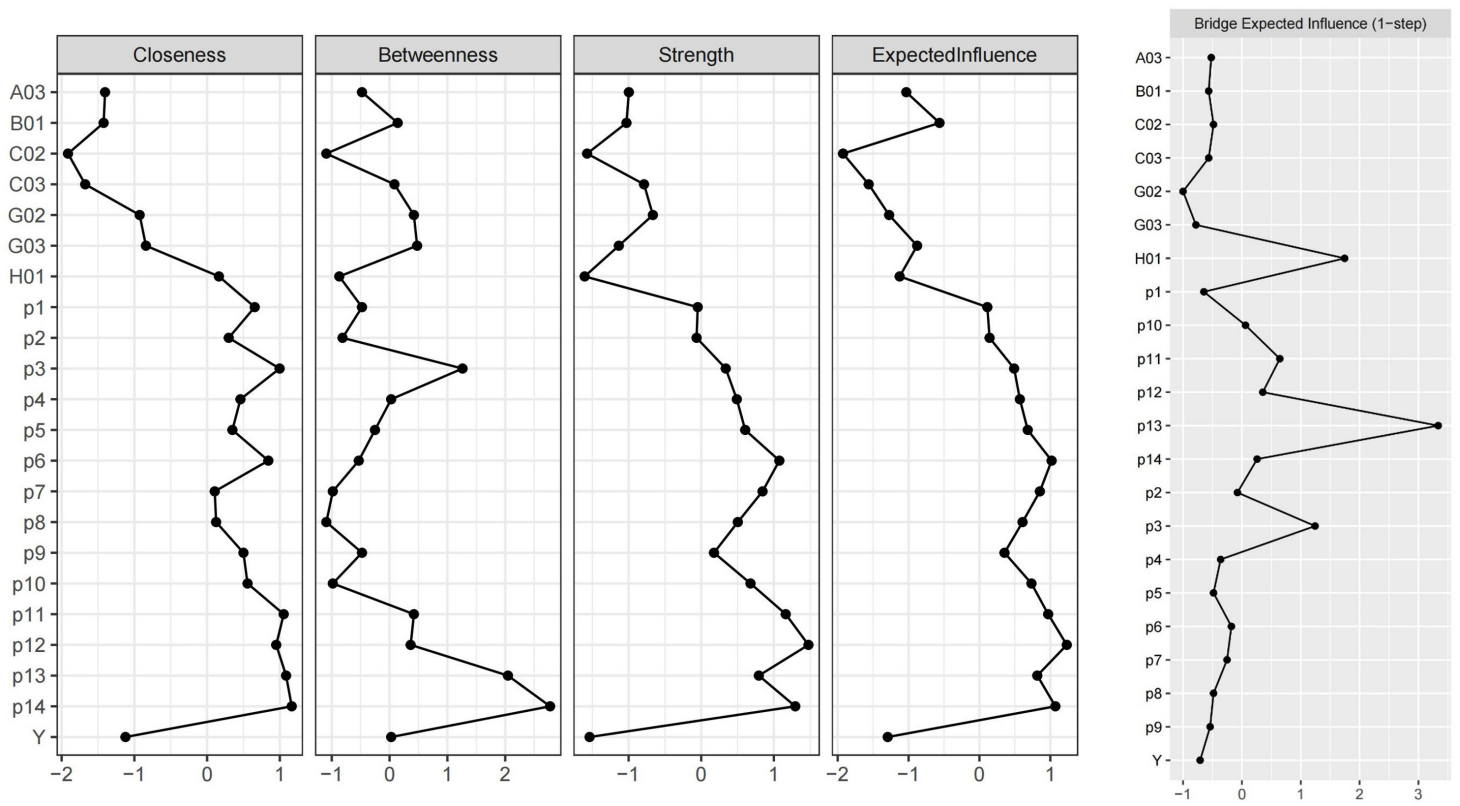
**Centrality and bridge indices of the network**. Note: The node names are shown in the legend of Fig. 1. Z scores were used when plotting the indices of the network.

Due to the presence of negative correlations in the network, we primarily relied on “expected influence” to analyze the centralities. As shown in Fig. 2, nodes “p12” (“failure”), “p14” (“confident”), “p6” (“satisfy”), and “p11” (“lonely and sad”) ranked among the top four. Among the top four nodes, only “p12” (“failure”) had a significant difference (uncorrected alpha = 0.05, 95% CI: [0.048, 0.283]) in expected influence with the fifth node “p7” (“helpless”). This indicated that “p12” (“failure”) was the most influential node in the entire network. Although the expected influence indices of “p14” (“confident”), “p6” (“satisfy”), and “p11” (“lonely and sad”) did not significantly exceed those of “p7” (“helpless”), they still held a relatively dominant position in the network. The results were similar to those of the network structure analyses with the exception of “p3” (“hopeless”) and “p13” (“life worth”). Based on “closeness” and “betweenness,” “p14” (“confident”) had the greatest sum of the shortest paths (significantly greater than “p1” (“happy of academic success”) and the other 15 nodes in terms of closeness; uncorrected alpha = 0.05, 95% CI: [0.0001, 0.0005]) and the most important bridge function (not significantly different from other nodes in statistics) among all the nodes. Considering PSI, NSI, and covariates as different communities, “p3” (“hopeless”), “p13” (“life worth”), and “H01” (“satisfaction of family members’ relationships”) were the most important linking nodes from the three communities. The bridge expected influence of “p13” (“life worth”) was significantly stronger than that of “p3” (“hopeless”) (uncorrected alpha = 0.05, 95% CI: [0.044, 0.238]) and the other 19 nodes. The bridge expected influence of “H01” (“satisfaction of family members’ relationships”) was significantly stronger than that of “p12” (“failure”) (uncorrected alpha = 0.05, 95% CI: [0.009, 0.195]) and the other 17 nodes. The bridge expected influence of “p3” (“hopeless”) was significantly stronger than that of “p10” (“suicide only option”) (uncorrected alpha = 0.05, 95% CI: [0.003, 0.180]) and the other 15 nodes. This illustrated that the three variables played a role as “bridges” between the communities. Fig. 2 displays the plots of the centrality bridge indices. The raw scores for all nodes’ centrality indices are provided in **Supplementary Table 2**.

### 3.4 Stability and Accuracy

**Fig. 3.**
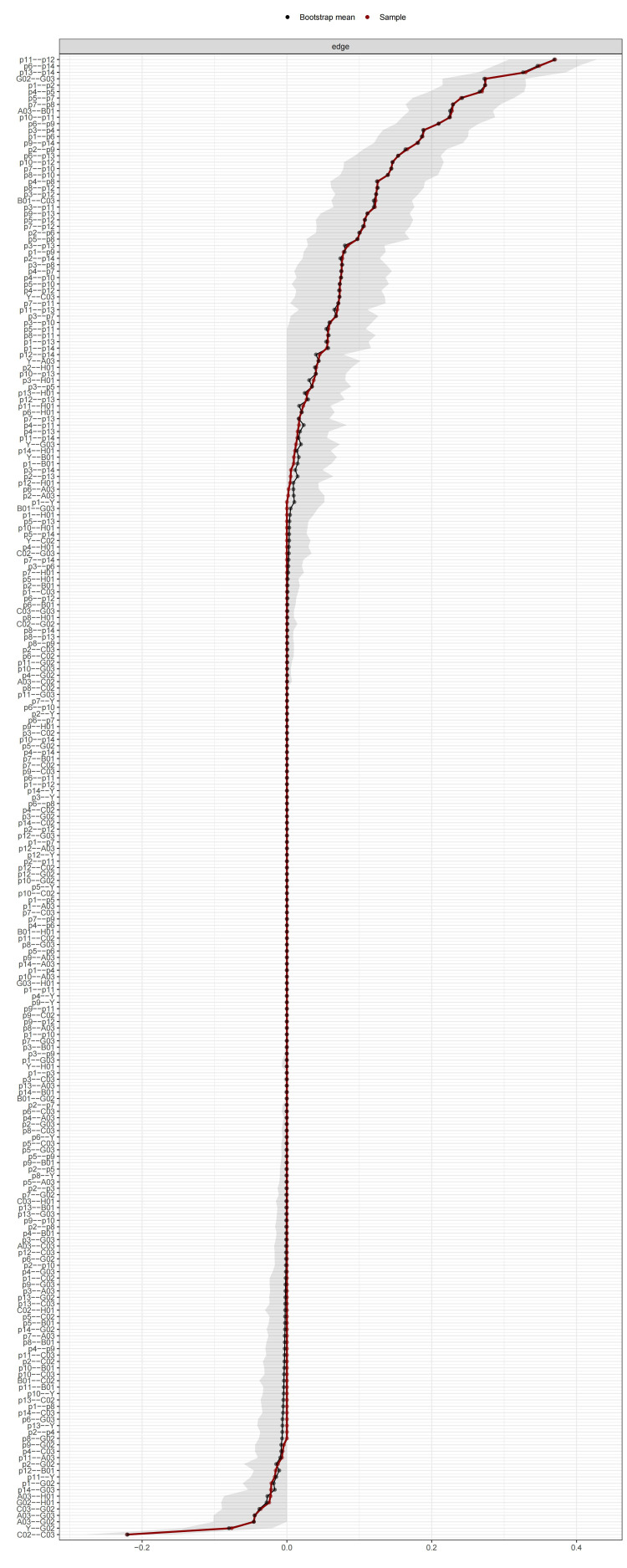
**Bootstrap means of the edge weights**.

**Fig. 4.**
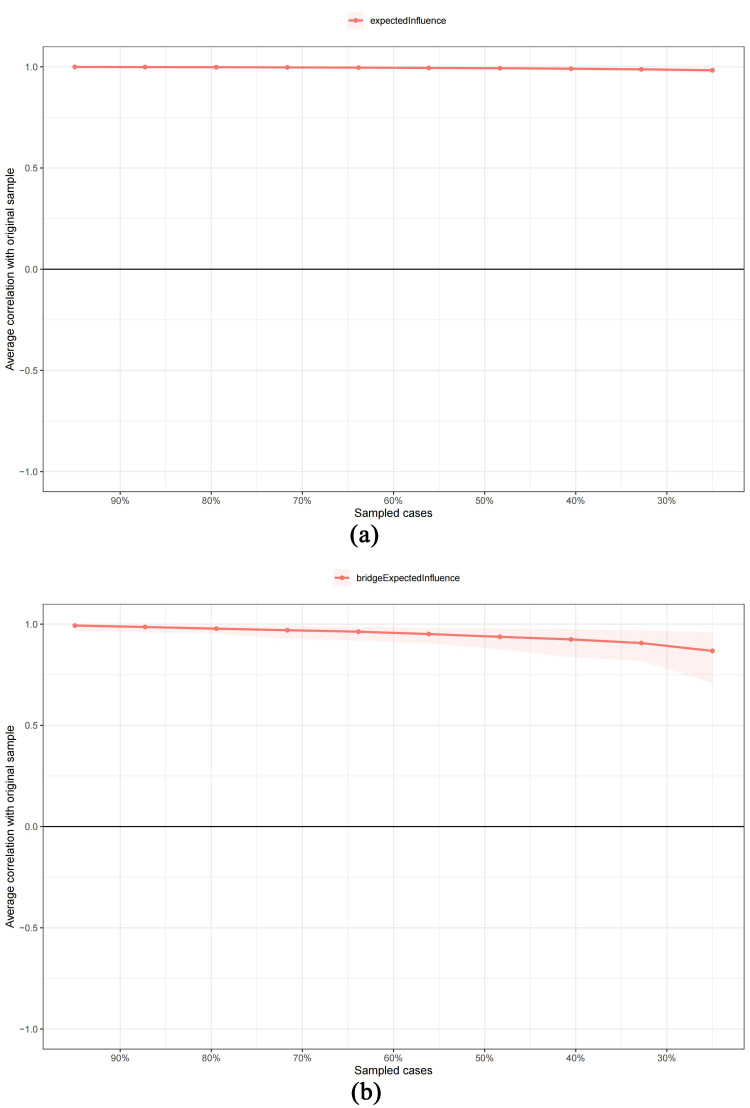
**Average correlations with the original sample**. Note: (a) plot of the expected influence of the whole network; (b) plot of the bridge expected influence.

We used the bootstrapping method with 2000 iterations to calculate the edge weight accuracy and the CS coefficient of expected influence and bridge expected influence. Fig. 3 demonstrated that the red line closely followed the black dots, indicating a strong correlation between the bootstrap means and the original sample edge weights. The CS coefficient for edge weight accuracy was 0.75, exceeding the recommended threshold of 0.5 [54]. This suggested that during the resampling process, even after removing 75% of the sample data, the correlation between the weights of the edges in the original network and the corresponding edges in the resampled network was still high (above 0.7) [54]. Both the CS coefficient and the visualized information confirmed the accuracy of the edge weight estimates. The CS coefficients for both expected influence and bridge expected influence were 0.75, exceeding the recommended threshold of 0.25 [54]. This paralleled the significance of the edge weight accuracy CS coefficient, suggesting that the estimates of expected influence and bridge expected influence were highly stable. Fig. 4 shows the average correlation of expected influence and bridge expected influence with the original sample across different sampling proportions. The red line remains above 0.7 across all sampling proportions, which could also reflect stable estimations. These results indicate that our centrality and bridge statistics are both accurate and stable.

### 3.5 Network Comparisons

**Fig. 5.**
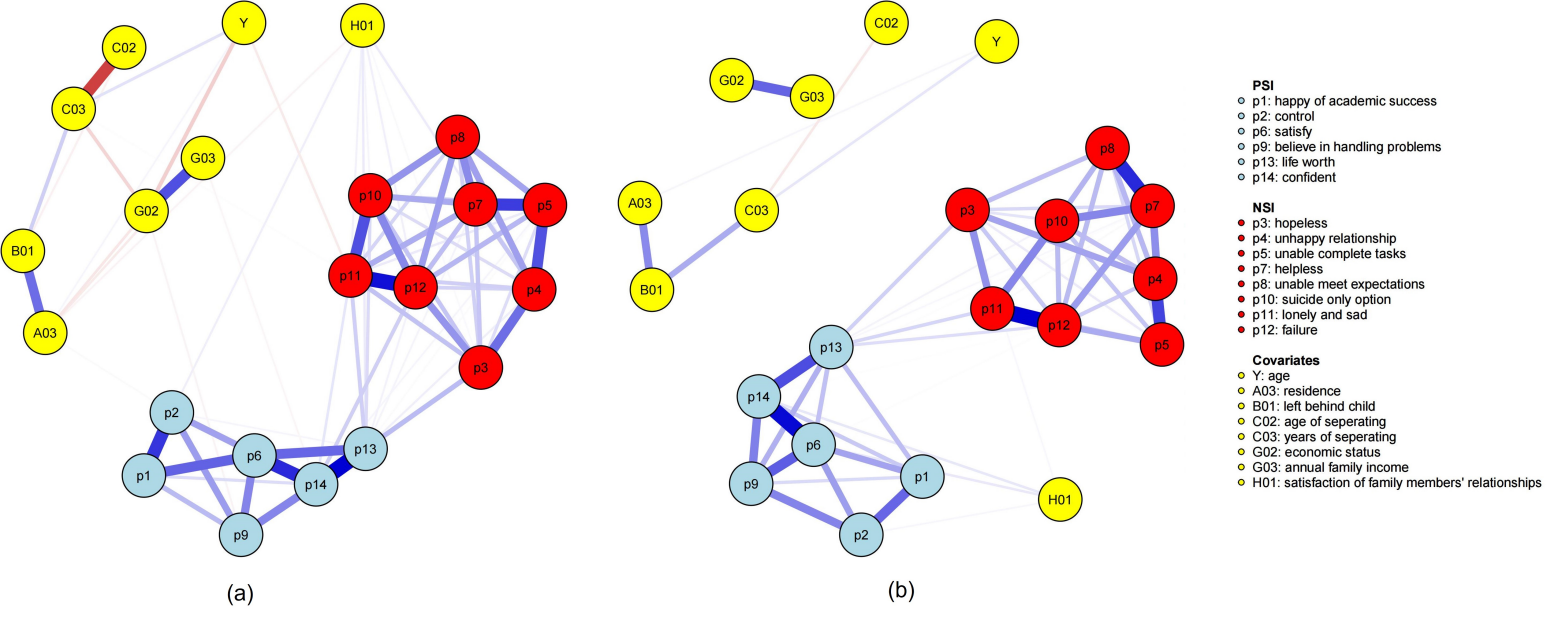
**Graphical structure of the female and male networks**. Note: (a) female network; (b) male network. Refer to Fig. 1 legend.

We only found differences in network structure between different genders (M = 0.236, *p *= 0.025). As shown in Fig. 5, in the female network, node “H01” (“satisfaction of family members’ relationships”) was correlated with both the covariates and the suicidal ideation communities, whereas in the male network, it separated from the covariates and was more closely related to suicidal ideation. Our finding suggests that among females, “H01” (“satisfaction of family members’ relationships”) was more closely associated with parental-economic factors and suicidal ideation symptoms. The values of the edge weights for the two networks are shown in **Supplementary Tables 3,4**. There was no significant difference in global strength between females and males (S = 0.864, *p* = 0.115). This finding indicates that the relationships among parental-economic factors and suicidal ideation symptoms have different patterns across genders. For only child status, there was no variance between only child and non-only child in terms of neither network structure (M = 0.148, *p *= 0.927) nor global strength (S = 0.606, *p* = 0.680).

## 4. Discussion

### 4.1 Summary of Findings

This study employed a network analysis approach to delve into the intricate relationships between parental-economic factors and suicidal ideation symptoms among LBC in underprivileged regions of China. Our results indicated that “hopeless”, “lonely and sad”, “failure”, and “satisfaction of family members’ relationships” exerted broad influence across the entire network, among which “failure” and “lonely and sad” were also strong. Additionally, “confident” acted as a pivotal bridge connecting all other network nodes. Meanwhile, “hopeless”, “life worth”, and “satisfaction of family members’ relationships” bridged the covariates and suicidal ideation, among which “satisfaction of family members’ relationships” was more linkable among females than males. These findings highlight the multifaceted nature of the relationships between parental-economic status and suicidal ideation, providing valuable insights into the underlying mechanisms that contribute to suicidal ideation in this vulnerable population.

### 4.2 Network Structure

Our network analysis revealed a clear and stable structure comprising three distinct communities: positive suicidal ideation, negative suicidal ideation, and covariates (sociodemographic, parental, economic status). This structural organization suggests that suicidal ideation is multifaceted and influenced by complex factors, rather than a single determinant. This finding aligns with those of previous studies, which have emphasized the multifaceted nature of suicidal ideation [2, 13]. Specifically, the strong links of “life worth—confident” and “satisfy—confident” indicate that these three symptoms form a subgroup and dominate within the PSI, among which “confident” is the core symptom. These findings agree with those of previous studies on the psychometric structure of the PANSI [35, 56]. However, “hopeless”, “lonely and sad”, and “failure” are more globally linked with other suicidal symptoms and influential factors in the network, partly consistent with the findings of Zhong *et al*. [57], who reported that failure and hopelessness were associated with suicidal ideation with the strongest edge weights. We have not yet found studies discussing positive or negative suicidal ideation among LBC from the perspective of symptom network. However, an existing study [58] reported that among college students, suicidal ideation symptoms form two communities and “hopeless” links globally in the network, which is similar to the findings of the present study. Nevertheless, the network structure alone does not permit us to conclude that interventions targeting hopelessness, distress, and feelings of loneliness and failure might be more effective than treatment in terms of confidence for children or adolescents. Further evidence regarding node strength is necessary to inform more targeted intervention strategies.

### 4.3 Expected Influence

The analysis of centrality indices reveals several key nodes with high expected influence within the network. Nodes representing “failure”, “confident”, “satisfy”, and “lonely and sad” emerged as the most influential, which is consistent with the implications of Yang *et al*. [16]. They investigated adolescents (not LBC) and reported that family functioning was related to suicidal ideation, whereas defeat and meaning of life played roles as mediators and moderators, respectively. Among these factors, the adaptation and partnership of family function, as well as views on defeat, align with our study. With respect to LBC, Xiao *et al*. [59] reported a positive association between the frequency of attachment with a migrating mother and self-harm. They inferred that the mother’s low educational level might account for this result. However, we suggest that more contact with migrating mothers may intensify feelings of loneliness in LBC, as they are unable to physically connect with their mother. The high expected influence of these nodes underscores their importance in driving suicidal ideation and highlights potential targets for intervention. The bridge expected influence analysis identified key variables that serve as crucial links between the covariate community and the suicidal ideation communities. Notably, “hopeless”, “life worth”, and “satisfaction of family members’ relationships” emerged as critical bridging variables. These findings suggest that interventions aimed at addressing feelings of hopelessness, enhancing perceptions of life worth, and improving family relationships may be particularly effective in mitigating suicidal ideation in LBC. The three variables are from three different communities, among which “life worth” has the strongest bridge expected influence. This illustrates the importance of life meaning among individuals with suicidal ideation symptoms. Moreover, Yang *et al*. [16] suggested that life meaning was a related factor of suicidal ideation but not a part of it, as they used a simpler scale (only four items) for measuring suicidal ideation. Among the various covariates, “satisfaction of family members’ relationships” was the most influential. This finding is consistent with prior studies emphasizing the role of family function, the family’s subjective atmosphere, and social support in buffering suicidal risk [1, 2, 60]. Compared with the other two variables, “hopeless” exhibited a weaker bridging role among communities. However, it was a directly related factor affecting the negative aspect of suicidal ideation which includes the key representations of thoughts. The important bridge role of hopelessness also aligns with another study [58] on the suicidal ideation of college students that applied network analysis. Among all the variables, hopelessness links other nodes widely but did not have a strong influence, whereas confidence had a strong influence within the whole network with relatively narrow linkages. This reminds us that when we want to achieve better intervention effects, we need to pay attention to both. SES did not emerge as a dominant factor in our network as in previous studies [18, 21, 22, 23, 24]. It may be associated with suicidal ideation via other variables, such as “satisfaction of family members’ relationships”. However, the SES nodes still had weak but nonzero connections with “confident” from the PSI community (see Fig. 1). In addition, “failure” and “lonely and sad” were both widely linked with and strongly influential on other nodes, which can help us to identify the key intervention targets. However, when we want to enhance preventive measures against suicidal ideation ahead of time, covariates need to be considered first, especially attitudes toward family relationships.

### 4.4 Network Comparisons

Network comparisons revealed gender differences in the patterns of relationships between parental-economic factors and suicidal ideation symptoms. Specifically, the node representing satisfaction with family relationships is more closely associated with suicidal ideation in females compared with males. This finding suggests that females may be more sensitive to family relationships, which is consistent with prior research on gender differences in suicidal ideation [61, 62]. Specifically, females experience more interpersonal conflicts and have a higher level of family self-concept. This underscores the importance of considering gender-specific risk factors and intervention strategies for LBC. Given that these studies were conducted in diverse cultural contexts, the findings further highlight the need for additional research to explore potential cultural and societal factors contributing to these gender differences.

### 4.5 Implications

Our findings have significant implications for preventive interventions for suicidal ideation among LBC in underprivileged regions of China. First, the identified key nodes and bridging variables provide targets for more tailored interventions aimed at reducing suicide risk. Second, the observed gender differences underscore the need for gender-sensitive approaches in suicide prevention efforts. Third, the network analysis approach employed in this study demonstrates its utility in revealing the complex relationships between multiple risk factors, offering a more nuanced understanding of suicidal ideation.

### 4.6 Limitations and Future Directions

While our study provides valuable insights, several limitations should be acknowledged. First, the cross-sectional design restricts our ability to draw causal inferences about the relationships between parental-economic factors and suicidal ideation. Second, our sample was drawn from a specific provincial population, limiting the generalizability of our findings. Third, while we controlled for several sociodemographic variables, other potentially important confounders (e.g., social relationships, prior trauma) were not assessed in this study. Finally, there may be biases in self-reported data because of its subjectivity, which can lead to unreliable inferences. Future studies could address the following: (1) longitudinal studies are needed to better understand the temporal dynamics of these relationships; (2) wider sampling is necessary from populations of other regions in China and even worldwide; (3) further research is needed to comprehensively account for more confounders of suicide risk; and (4) more objective suicidal ideation measurements should be developed.

## 5. Conclusions

In conclusion, our network analysis highlights the complex interplay between parental-economic factors and suicidal ideation symptoms among LBC in underprivileged regions of China. The identified key nodes and bridging variables offer valuable targets for tailored interventions aimed at reducing suicide risk in this vulnerable population. Future research should build upon these findings to develop and evaluate evidence-based preventive intervention strategies.

## Data Availability

All the data and materials are available from the corresponding author upon request.

## Abbreviations

BSI, Beck’s Scale for Suicide Ideation; CI, confidence interval; CPHG, Children and Adolescents Project of China Group; CS, correlation stability; FSM, family stress model; GGM, Gaussian graphical model; GLASSO, graphic least absolute shrinkage and selection operator; LBC, left-behind children; NSI, negative suicidal ideation; NSSI, non-suicidal self-injury; PANSI, Positive and Negative Suicidal Ideation Inventory; PANSI-C, Positive and Negative Suicide Ideation Inventory-Chinese Version ; PCM, partial correlation model; PSI, positive suicidal ideation; SD, standard deviation; SES, socioeconomic status.

## Supplementary Material

Supplementary material associated with this article can be found, in the online version, at https://doi.org/10.31083/AP43496.
