# Supplementary material for: Network Analysis of Parental-Economic Factors and Symptoms of Suicidal Ideation Among Left-Behind Children in Unprivileged Regions in China
Source: Alpha Psychiatry. 2025 Jun 10;26(3):43496. doi: 10.31083/AP43496 (PMC12231424; doi:10.31083/AP43496)
Supplement: Supplementary file 1 [file 2757-8038-26-3-43496-s1.docx]

Supplementary Table 1. Edge weights list of the whole network.

|  | p1 | p2 | p3 | p4 | p5 | p6 | p7 | p8 | p9 | p10 | p11 | p12 | p13 | p14 | Y | A03 | B01 | C02 | C03 | G02 | G03 | H01 |
| --- | --- | --- | --- | --- | --- | --- | --- | --- | --- | --- | --- | --- | --- | --- | --- | --- | --- | --- | --- | --- | --- | --- |
| p1 |  |  |  |  |  |  |  |  |  |  |  |  |  |  |  |  |  |  |  |  |  |  |
| p2 | 0.27 |  |  |  |  |  |  |  |  |  |  |  |  |  |  |  |  |  |  |  |  |  |
| p3 | 0.00 | 0.00 |  |  |  |  |  |  |  |  |  |  |  |  |  |  |  |  |  |  |  |  |
| p4 | 0.00 | 0.00 | 0.19 |  |  |  |  |  |  |  |  |  |  |  |  |  |  |  |  |  |  |  |
| p5 | 0.00 | 0.00 | 0.03 | 0.27 |  |  |  |  |  |  |  |  |  |  |  |  |  |  |  |  |  |  |
| p6 | 0.19 | 0.10 | 0.00 | 0.00 | 0.00 |  |  |  |  |  |  |  |  |  |  |  |  |  |  |  |  |  |
| p7 | 0.00 | 0.00 | 0.07 | 0.08 | 0.24 | 0.00 |  |  |  |  |  |  |  |  |  |  |  |  |  |  |  |  |
| p8 | 0.00 | 0.00 | 0.08 | 0.13 | 0.10 | 0.00 | 0.23 |  |  |  |  |  |  |  |  |  |  |  |  |  |  |  |
| p9 | 0.08 | 0.17 | 0.00 | 0.00 | 0.00 | 0.21 | 0.00 | 0.00 |  |  |  |  |  |  |  |  |  |  |  |  |  |  |
| p10 | 0.00 | 0.00 | 0.06 | 0.07 | 0.07 | 0.00 | 0.14 | 0.14 | 0.00 |  |  |  |  |  |  |  |  |  |  |  |  |  |
| p11 | 0.00 | 0.00 | 0.12 | 0.02 | 0.06 | 0.00 | 0.07 | 0.06 | 0.00 | 0.23 |  |  |  |  |  |  |  |  |  |  |  |  |
| p12 | 0.00 | 0.00 | 0.12 | 0.07 | 0.11 | 0.00 | 0.11 | 0.12 | 0.00 | 0.15 | 0.37 |  |  |  |  |  |  |  |  |  |  |  |
| p13 | 0.06 | 0.01 | 0.08 | 0.01 | 0.00 | 0.15 | 0.02 | 0.00 | 0.11 | 0.04 | 0.07 | 0.03 |  |  |  |  |  |  |  |  |  |  |
| p14 | 0.06 | 0.08 | 0.01 | 0.00 | 0.00 | 0.35 | 0.00 | 0.00 | 0.18 | 0.00 | 0.01 | 0.05 | 0.33 |  |  |  |  |  |  |  |  |  |
| Y | 0.00 | 0.00 | 0.00 | 0.00 | 0.00 | 0.00 | 0.00 | 0.00 | 0.00 | 0.00 | -0.02 | 0.00 | 0.00 | 0.00 |  |  |  |  |  |  |  |  |
| A03 | 0.00 | 0.00 | 0.00 | 0.00 | 0.00 | 0.00 | 0.00 | 0.00 | 0.00 | 0.00 | -0.01 | 0.00 | 0.00 | 0.00 | 0.04 |  |  |  |  |  |  |  |
| B01 | 0.01 | 0.00 | 0.00 | 0.00 | 0.00 | 0.00 | 0.00 | 0.00 | 0.00 | 0.00 | 0.00 | -0.02 | 0.00 | 0.00 | 0.01 | 0.23 |  |  |  |  |  |  |
| C02 | 0.00 | 0.00 | 0.00 | 0.00 | 0.00 | 0.00 | 0.00 | 0.00 | 0.00 | 0.00 | 0.00 | 0.00 | 0.00 | 0.00 | 0.00 | 0.00 | 0.00 |  |  |  |  |  |
| C03 | 0.00 | 0.00 | 0.00 | -0.01 | 0.00 | 0.00 | 0.00 | 0.00 | 0.00 | 0.00 | 0.00 | 0.00 | 0.00 | 0.00 | 0.07 | 0.00 | 0.12 | -0.22 |  |  |  |  |
| G02 | -0.02 | -0.01 | 0.00 | 0.00 | 0.00 | 0.00 | 0.00 | 0.00 | 0.00 | 0.00 | 0.00 | 0.00 | 0.00 | 0.00 | -0.08 | -0.05 | 0.00 | 0.00 | -0.04 |  |  |  |
| G03 | 0.00 | 0.00 | 0.00 | 0.00 | 0.00 | 0.00 | 0.00 | 0.00 | 0.00 | 0.00 | 0.00 | 0.00 | 0.00 | -0.02 | 0.01 | -0.04 | 0.00 | 0.00 | 0.00 | 0.27 |  |  |
| H01 | 0.00 | 0.04 | 0.04 | 0.00 | 0.00 | 0.02 | 0.00 | 0.00 | 0.00 | 0.00 | 0.02 | 0.00 | 0.03 | 0.01 | 0.00 | -0.02 | 0.00 | 0.00 | 0.00 | -0.02 | 0.00 |  |

Note: p1, happy of academic success; p2, control; p3, hopeless; p4, unhappy relationship; p5, unable complete tasks; p6, satisfy; p7, helpless; p8, unable meet expectations; p9, believe in handling problems; p10, suicide only option; p11, lonely and sad; p12, failure; p13, life worth; p14, confident; Y, age; A03, residence; B01, left behind child; C02, age of separating; C03, years of separating; G02, economic status; G03, annual family income; H01, satisfication of family members’ relationships.

Supplementary Table 2. The raw scores of all nodes’ centrality indexes.

| Nodes | Closeness | Betweenness | Strength | Expected influence | Bridge expected influence |
| --- | --- | --- | --- | --- | --- |
| p1 | 0.001322117 | 22 | 0.681975584 | 0.639098534 | -0.012033007 |
| p2 | 0.001241401 | 10 | 0.677237184 | 0.652439275 | 0.029860755 |
| p3 | 0.001399338 | 84 | 0.798805173 | 0.798805173 | 0.126787027 |
| p4 | 0.001277791 | 40 | 0.844919343 | 0.832972102 | 0.008862336 |
| p5 | 0.001252771 | 30 | 0.879881337 | 0.879881337 | 0 |
| p6 | 0.001364278 | 20 | 1.022335874 | 1.022335874 | 0.022313457 |
| p7 | 0.001198016 | 4 | 0.952224872 | 0.952224872 | 0.016882204 |
| p8 | 0.001202205 | 0 | 0.849031895 | 0.849031895 | 0 |
| p9 | 0.001287036 | 22 | 0.749880309 | 0.741426425 | -0.004226942 |
| p10 | 0.001299832 | 4 | 0.902094061 | 0.902094061 | 0.039917148 |
| p11 | 0.001412075 | 54 | 1.048890625 | 1.001621344 | 0.082787241 |
| p12 | 0.001388797 | 52 | 1.143501647 | 1.112619204 | 0.06150026 |
| p13 | 0.001419448 | 112 | 0.936732932 | 0.936732932 | 0.280523377 |
| p14 | 0.00143698 | 138 | 1.088564952 | 1.044580396 | 0.054443511 |
| Y | 0.000920874 | 40 | 0.231199672 | 0.045222834 | -0.016714684 |
| A03 | 0.000857484 | 22 | 0.394965387 | 0.156062473 | -0.002829073 |
| B01 | 0.000853116 | 44 | 0.384967435 | 0.354084993 | -0.006035704 |
| C02 | 0.000742444 | 0 | 0.220988795 | -0.220988795 | 0 |
| C03 | 0.000795924 | 42 | 0.458204634 | -0.06790597 | -0.005973621 |
| G02 | 0.000964942 | 54 | 0.49502597 | 0.05373495 | -0.038064421 |
| G03 | 0.000984349 | 56 | 0.353129579 | 0.220635314 | -0.021992278 |
| H01 | 0.001210942 | 8 | 0.210801945 | 0.116644034 | 0.163722989 |

Note: Meanings of the nodes were listed in the note of Table 1S.

Supplementary Table 3. Edge weights list of the female network.

|  | p1 | p2 | p3 | p4 | p5 | p6 | p7 | p8 | p9 | p10 | p11 | p12 | p13 | p14 | Y | A03 | B01 | C02 | C03 | G02 | G03 | H01 |
| --- | --- | --- | --- | --- | --- | --- | --- | --- | --- | --- | --- | --- | --- | --- | --- | --- | --- | --- | --- | --- | --- | --- |
| p1 |  |  |  |  |  |  |  |  |  |  |  |  |  |  |  |  |  |  |  |  |  |  |
| p2 | 0.28 |  |  |  |  |  |  |  |  |  |  |  |  |  |  |  |  |  |  |  |  |  |
| p3 | 0.00 | 0.00 |  |  |  |  |  |  |  |  |  |  |  |  |  |  |  |  |  |  |  |  |
| p4 | 0.00 | 0.00 | 0.20 |  |  |  |  |  |  |  |  |  |  |  |  |  |  |  |  |  |  |  |
| p5 | 0.00 | 0.00 | 0.04 | 0.24 |  |  |  |  |  |  |  |  |  |  |  |  |  |  |  |  |  |  |
| p6 | 0.21 | 0.06 | 0.00 | 0.00 | 0.00 |  |  |  |  |  |  |  |  |  |  |  |  |  |  |  |  |  |
| p7 | 0.00 | 0.00 | 0.08 | 0.08 | 0.26 | 0.00 |  |  |  |  |  |  |  |  |  |  |  |  |  |  |  |  |
| p8 | 0.00 | 0.00 | 0.06 | 0.13 | 0.12 | 0.00 | 0.16 |  |  |  |  |  |  |  |  |  |  |  |  |  |  |  |
| p9 | 0.09 | 0.13 | 0.00 | 0.00 | 0.00 | 0.17 | 0.00 | 0.00 |  |  |  |  |  |  |  |  |  |  |  |  |  |  |
| p10 | 0.00 | 0.00 | 0.08 | 0.05 | 0.07 | 0.00 | 0.12 | 0.15 | 0.00 |  |  |  |  |  |  |  |  |  |  |  |  |  |
| p11 | 0.00 | 0.00 | 0.08 | 0.07 | 0.03 | 0.00 | 0.11 | 0.06 | 0.00 | 0.24 |  |  |  |  |  |  |  |  |  |  |  |  |
| p12 | 0.00 | 0.00 | 0.14 | 0.06 | 0.10 | 0.00 | 0.09 | 0.13 | 0.00 | 0.16 | 0.33 |  |  |  |  |  |  |  |  |  |  |  |
| p13 | 0.02 | 0.01 | 0.09 | 0.02 | 0.00 | 0.21 | 0.01 | 0.00 | 0.11 | 0.05 | 0.07 | 0.02 |  |  |  |  |  |  |  |  |  |  |
| p14 | 0.05 | 0.13 | 0.00 | 0.00 | 0.01 | 0.29 | 0.00 | 0.00 | 0.17 | 0.00 | 0.03 | 0.06 | 0.35 |  |  |  |  |  |  |  |  |  |
| Y | 0.00 | 0.00 | 0.00 | 0.00 | 0.00 | 0.00 | 0.00 | 0.00 | 0.00 | 0.00 | -0.03 | 0.00 | 0.00 | 0.00 |  |  |  |  |  |  |  |  |
| A03 | 0.00 | 0.01 | 0.00 | 0.00 | 0.00 | 0.00 | 0.00 | 0.00 | 0.00 | 0.00 | 0.00 | 0.00 | 0.00 | 0.00 | 0.01 |  |  |  |  |  |  |  |
| B01 | 0.00 | 0.00 | 0.00 | 0.00 | 0.00 | 0.00 | 0.00 | 0.00 | 0.00 | 0.00 | 0.00 | 0.00 | 0.00 | 0.00 | 0.00 | 0.20 |  |  |  |  |  |  |
| C02 | 0.00 | 0.00 | 0.00 | 0.00 | 0.00 | 0.00 | 0.00 | 0.00 | 0.00 | 0.00 | 0.00 | 0.00 | 0.00 | 0.00 | 0.00 | 0.00 | -0.02 |  |  |  |  |  |
| C03 | 0.00 | 0.00 | 0.00 | 0.00 | 0.00 | 0.00 | 0.00 | 0.00 | 0.00 | 0.00 | 0.00 | 0.00 | 0.00 | 0.00 | 0.04 | 0.00 | 0.06 | -0.26 |  |  |  |  |
| G02 | 0.00 | 0.00 | 0.00 | 0.00 | 0.00 | 0.00 | 0.00 | 0.00 | -0.02 | 0.00 | 0.00 | 0.00 | 0.00 | 0.00 | -0.07 | -0.03 | 0.00 | 0.00 | -0.06 |  |  |  |
| G03 | 0.00 | 0.00 | 0.00 | 0.00 | 0.00 | 0.00 | 0.00 | 0.00 | 0.00 | 0.00 | 0.00 | 0.00 | 0.00 | -0.01 | 0.00 | -0.03 | 0.00 | 0.00 | 0.00 | 0.24 |  |  |
| H01 | 0.00 | 0.02 | 0.01 | 0.03 | 0.00 | 0.00 | 0.00 | 0.00 | 0.00 | 0.01 | 0.02 | 0.02 | 0.03 | 0.00 | 0.00 | -0.01 | 0.00 | 0.00 | 0.00 | 0.00 | 0.00 |  |

Note: Meanings of the nodes were listed in the note of Table 1S.

Supplementary Table 4. Edge weights list of the male network.

|  | p1 | p2 | p3 | p4 | p5 | p6 | p7 | p8 | p9 | p10 | p11 | p12 | p13 | p14 | Y | A03 | B01 | C02 | C03 | G02 | G03 | H01 |
| --- | --- | --- | --- | --- | --- | --- | --- | --- | --- | --- | --- | --- | --- | --- | --- | --- | --- | --- | --- | --- | --- | --- |
| p1 |  |  |  |  |  |  |  |  |  |  |  |  |  |  |  |  |  |  |  |  |  |  |
| p2 | 0.23 |  |  |  |  |  |  |  |  |  |  |  |  |  |  |  |  |  |  |  |  |  |
| p3 | 0.00 | 0.00 |  |  |  |  |  |  |  |  |  |  |  |  |  |  |  |  |  |  |  |  |
| p4 | 0.00 | 0.00 | 0.14 |  |  |  |  |  |  |  |  |  |  |  |  |  |  |  |  |  |  |  |
| p5 | 0.00 | 0.00 | 0.03 | 0.29 |  |  |  |  |  |  |  |  |  |  |  |  |  |  |  |  |  |  |
| p6 | 0.14 | 0.15 | 0.00 | 0.00 | 0.00 |  |  |  |  |  |  |  |  |  |  |  |  |  |  |  |  |  |
| p7 | 0.00 | 0.00 | 0.06 | 0.08 | 0.17 | 0.00 |  |  |  |  |  |  |  |  |  |  |  |  |  |  |  |  |
| p8 | 0.00 | 0.00 | 0.10 | 0.11 | 0.06 | 0.00 | 0.33 |  |  |  |  |  |  |  |  |  |  |  |  |  |  |  |
| p9 | 0.06 | 0.19 | 0.00 | 0.00 | 0.00 | 0.25 | 0.00 | 0.00 |  |  |  |  |  |  |  |  |  |  |  |  |  |  |
| p10 | 0.00 | 0.00 | 0.04 | 0.10 | 0.09 | 0.00 | 0.18 | 0.11 | 0.00 |  |  |  |  |  |  |  |  |  |  |  |  |  |
| p11 | 0.00 | 0.00 | 0.17 | 0.00 | 0.09 | 0.00 | 0.01 | 0.07 | 0.00 | 0.18 |  |  |  |  |  |  |  |  |  |  |  |  |
| p12 | 0.00 | 0.00 | 0.08 | 0.08 | 0.13 | 0.00 | 0.15 | 0.10 | 0.00 | 0.10 | 0.39 |  |  |  |  |  |  |  |  |  |  |  |
| p13 | 0.10 | 0.00 | 0.07 | 0.00 | 0.00 | 0.09 | 0.01 | 0.00 | 0.12 | 0.02 | 0.06 | 0.05 |  |  |  |  |  |  |  |  |  |  |
| p14 | 0.08 | 0.00 | 0.00 | 0.00 | 0.00 | 0.37 | 0.00 | 0.00 | 0.19 | 0.00 | 0.00 | 0.00 | 0.27 |  |  |  |  |  |  |  |  |  |
| Y | 0.00 | 0.00 | 0.00 | 0.00 | 0.00 | 0.00 | 0.00 | 0.00 | 0.00 | 0.00 | 0.00 | 0.00 | 0.00 | 0.00 |  |  |  |  |  |  |  |  |
| A03 | 0.00 | 0.00 | 0.00 | 0.00 | 0.00 | 0.00 | 0.00 | 0.00 | 0.00 | 0.00 | 0.00 | 0.00 | 0.00 | 0.00 | 0.01 |  |  |  |  |  |  |  |
| B01 | 0.00 | 0.00 | 0.00 | 0.00 | 0.00 | 0.00 | 0.00 | 0.00 | 0.00 | 0.00 | 0.00 | 0.00 | 0.00 | 0.00 | 0.00 | 0.18 |  |  |  |  |  |  |
| C02 | 0.00 | 0.00 | 0.00 | 0.00 | 0.00 | 0.00 | 0.00 | 0.00 | 0.00 | 0.00 | 0.00 | 0.00 | 0.00 | 0.00 | 0.00 | 0.00 | 0.00 |  |  |  |  |  |
| C03 | 0.00 | 0.00 | 0.00 | 0.00 | 0.00 | 0.00 | 0.00 | 0.00 | 0.00 | 0.00 | 0.00 | 0.00 | 0.00 | 0.00 | 0.04 | 0.00 | 0.13 | -0.04 |  |  |  |  |
| G02 | 0.00 | 0.00 | 0.00 | 0.00 | 0.00 | 0.00 | 0.00 | 0.00 | 0.00 | 0.00 | 0.00 | 0.00 | 0.00 | 0.00 | 0.00 | 0.00 | 0.00 | 0.00 | 0.00 |  |  |  |
| G03 | 0.00 | 0.00 | 0.00 | 0.00 | 0.00 | 0.00 | 0.00 | 0.00 | 0.00 | 0.00 | 0.00 | 0.00 | 0.00 | 0.00 | 0.00 | 0.00 | 0.00 | 0.00 | 0.00 | 0.24 |  |  |
| H01 | 0.00 | 0.01 | 0.01 | 0.00 | 0.00 | 0.03 | 0.00 | 0.00 | 0.00 | 0.00 | 0.00 | 0.00 | 0.00 | 0.03 | 0.00 | 0.00 | 0.00 | 0.00 | 0.00 | 0.00 | 0.00 |  |

Note: Meanings of the nodes were listed in the note of Table 1S.
